# Color constancy in real-world settings

**DOI:** 10.1167/jov.24.2.12

**Published:** 2024-02-27

**Authors:** Karl R. Gegenfurtner, David Weiss, Marina Bloj

**Affiliations:** 1 Department of Psychology, Giessen University, Giessen, Germany; 2 Faculty of Health Studies, University of Bradford, Bradford, UK

**Keywords:** color constancy, natural environments, illumination changes, natural task

## Abstract

Color constancy denotes the ability to assign a particular and stable color percept to an object, irrespective of its surroundings and illumination. The light reaching the eye confounds illumination and spectral reflectance of the object, making the recovery of constant object color an ill-posed problem. How good the visual system is at accomplishing this task is still a matter of heated debate, despite more than a 100 years of research. Depending on the laboratory task and the specific cues available to observers, color constancy was found to be at levels ranging between 15% and 80%, which seems incompatible with the relatively stable color appearance of objects around us and the consistent usage of color names in real life. Here, we show close-to-perfect color constancy using real objects in a natural task and natural environmental conditions, chosen to mimic the role of color constancy in everyday life. Participants had to identify the color of a (non-present) item familiar to them in an office room under five different experimental illuminations. They mostly selected the same colored Munsell chip as their match to the absent object, even though the light reaching the eye in each case differed substantially. Our results demonstrate that color constancy under ideal conditions in the real world can indeed be exceptionally good. We found it to be as good as visual memory permits and not generally compromised by sensory uncertainty.

## Introduction

Perceptual constancies are the workhorse of our sensory abilities. In vision, the stimulation on the retina is extremely variable with respect to size, form, speed, and wavelength. Yet, we do perceive a stable world where, for example, an object does not appear to change when we walk past it, even though changes in distance, projection, eccentricity, and illumination might lead to a vastly different stimulation of our visual system. For many of these constancies, it is known that the compensation achieved by our visual system depends on the richness of contextual cues that are available. This has been shown very elegantly for size constancy in the classic experiments of Holway and Boring (1941), and similar findings have emerged for other constancies (see Epstein, 1977; Walsh & Kulikowski, 1998).

Color constancy seems to be a notable exception, and the degree to which humans achieve color constancy is still heavily debated (for reviews, see Foster, 2003; Foster, 2011). On the one hand, some researchers argue that the problem of color constancy is intractable mainly due to metamerism (Logvinenko, Funt, Mirzaei, & Tokunaga, 2015; Witzel, van Alphen, Godau, & O'Regan, 2016), and several empirical studies have observed rather low levels of compensation, around 20% (Arend & Reeves, 1986; Tiplitz, Blackwell, & Buchsbaum, 1988; Valberg & Lange-Malecki, 1990). On the other hand, experiments have shown that, as cues are added to the visual stimulus, color constancy increases to levels of about 80% (Kraft & Brainard, 1999). Indeed, over several decades the degree of color constancy measured experimentally seems to have steadily risen (Foster, 2011; Witzel & Gegenfurtner, 2018).

At least partly, this controversy seems to be due to the introduction of very strict methods to quantify color constancy in terms of a single number. A hundred years ago, nobody doubted that there would be a constancy of color to a very high degree (Gelb, 1929; Katz, 1911; Koffka, 1932). The main question was how this was achieved. von Helmholtz (1910) proposed the influence of unconscious inferences, possibly supported by low-level sensory adaptation (von Kries, 1923). The view of Hering (1920) is quite close to current thinking. He proposed low-level effects of adaptation and simultaneous contrast, supported by high-level effects of memory color: the known color of some objects. Empirical studies by Katz (1911) found that a large field of view and a structured scene led to very good constancy. Helson (1938) and Helson and Jeffers (1940) agreed with this view but observed that constancy would be poorer under highly saturated chromatic illuminations.

Many of the 20th-century studies observing low degrees of constancy have used asymmetric matching techniques with two different illuminants simulated on a CRT monitor displaying a range of flat, matte surfaces. Observers have to match the color of two test objects in these different regions (Arend & Reeves, 1986). This allows testing objects with arbitrary colors, but it also limits the degree of adaptation that is possible, and local contrast becomes basically the only cue that is available for achieving color constancy. Constancy in these paradigms has generally been poor, but experiments with more immersive environments and illumination gradients have brought the degree of constancy up to 60% (Brainard, Brunt, & Speigle, 1997). The degree of immersion can also be increased by using achromatic matching, sequential matching, or color categorization under a single, full-field illuminant. In many of these studies, relatively high levels of constancy at or above 80% have been found (Brainard, 1998; Foster, Amano, & Nascimento, 2001; Hansen, Walter, & Gegenfurtner, 2007; Olkkonen, Hansen, & Gegenfurtner, 2009; Olkkonen, Witzel, Hansen, & Gegenfurtner, 2010; Smithson & Zaidi, 2004). However, there are also drawbacks to these paradigms. In achromatic matching, the observer makes neutral settings under different illuminations. Therefore, the only “color” that is ever adjusted is gray. In categorization and naming tasks, there is a severe limit to the resolution, because only a limited number of categories or names can be used. What is missing from the literature is a paradigm that mimics color constancy in everyday life. The selection paradigm, where observers have to select a colored target seen under illuminant A under a different illuminant B, comes closer to real life, but so far it has been mainly used in asymmetric viewing contexts (Bramwell & Hurlbert, 1996; Radonjic, Cottaris, & Brainard, 2015a; Radonjic, Cottaris, & Brainard, 2015b; Zaidi & Bostic, 2008). Although the degree of constancy was higher than in standard matching tasks, it seems that constancy was still limited by factors related to the asymmetric matching paradigm.

In everyday life, we take it for granted that objects “have” a color. From early childhood on, we regularly use color terms to describe objects (Bornstein, 1985; Franklin & Davies, 2004). We say that a shirt “is” green, for example, and not that the shirt “looks greenish under this particular lighting.” Our high, innate expectation of color as a common and reliable object identifier became clear in February 2015 when what seemed to be the whole world became irritated by looking at the same picture but seeing the portrayed object as different colors: the well-publicized blue–black/white–gold dress conundrum (Aston & Hurlbert, 2017; Brainard & Hurlbert, 2015; Gegenfurtner, Bloj, & Toscani, 2015; Uchikawa, Morimoto, & Matsumoto, 2017; Lafer-Sousa, Hermann, & Conway, 2015; Schlaffke et al., 2015; Toscani, Gegenfurtner, & Dörschner, 2017; Wallisch, 2017; Winkler, Spillmann, Werner, & Webster, 2015; Witzel, Racey, & O'Regan, 2017).

Given the low levels of color constancy observed in some experiments and the variability between different observers and tasks (e.g., Arend & Reeves, 1986; Radonjic & Brainard, 2016), one would expect these inconsistencies in color identification across different observers to happen frequently. This does not seem to be the case, hence the uproar in 2015. In order to be able to use color terms consistently and persistently, colors have to be remembered. However, solely remembering colors would not be enough to recognize our favorite shirt outside in bright sunlight and inside in the office under artificial light. From a physical point of view, the spectral distribution of the light that reaches our eyes from the shirt in these situations is very different, as are the ensuing excitations of the cone photoreceptors in the retina and yet, indoors and outside, the shirt seems to mostly appear the same color to us.

Our visual system seems able to compensate for such illumination and surround changes (for reviews, see Brainard & Radonjic, 2014; Foster, 2011; Hurlbert, 2007; Olkkonen & Ekroll, 2016; Smithson, 2005) and enables us to perceive the environment as relatively stable with respect to color. In other words, we perceive objects as color constant.

**Figure 1.**
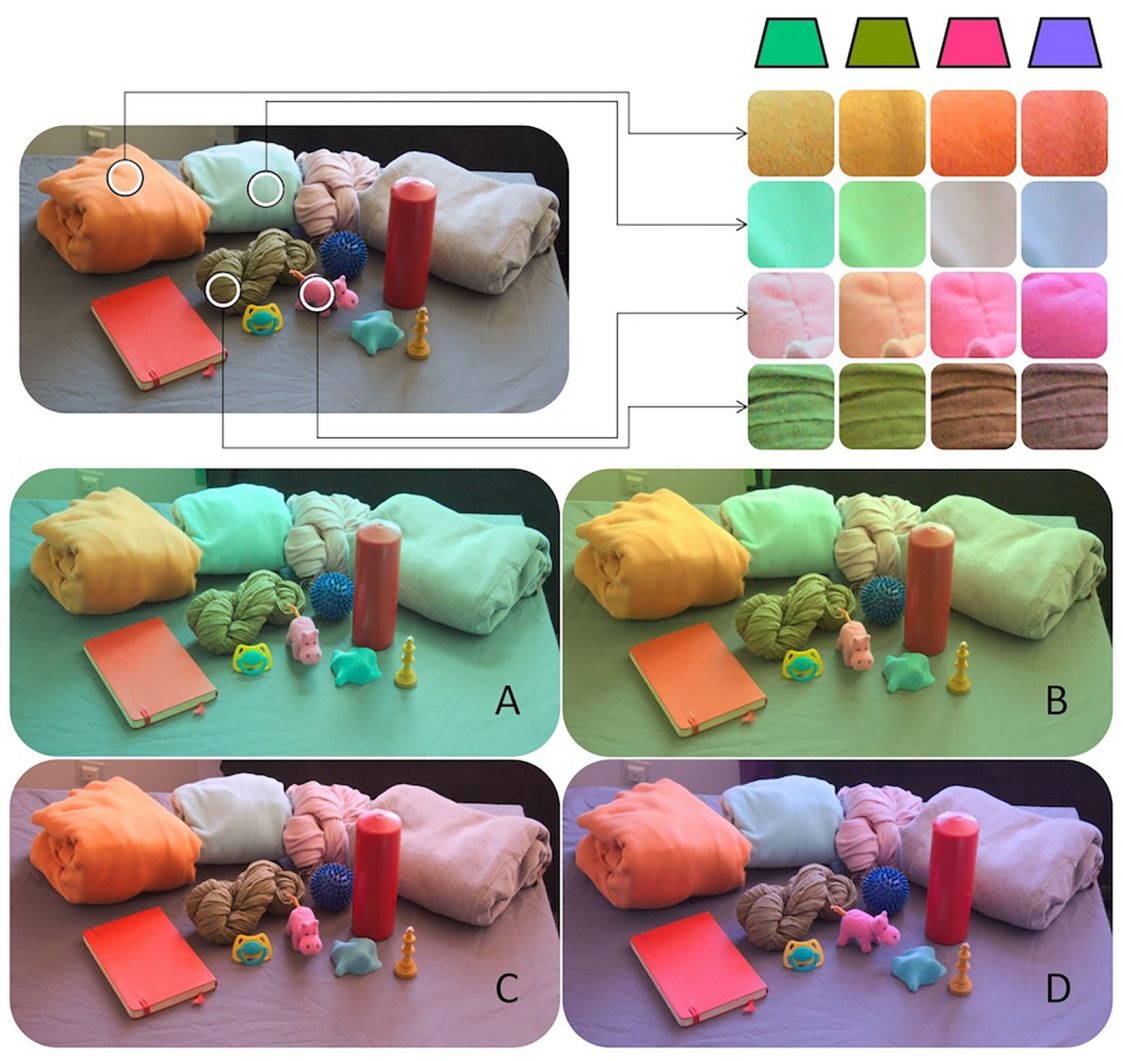
Personal objects brought for the study by participants shown under neutral daylight illumination (top left) and the four experimental filter conditions (**A**–**D**). In the top right, we show details of several objects under the four colorful illuminants to illustrate how different the reflected wavelength composition and cone excitations are under each illumination. These images were taken with a Nikon D70 digital camera with the white balancing turned off. They were not colorimetrically calibrated and only serve illustration purposes. The objects were never seen by the observers during the experiments.

Figure 1 illustrates why this is a major achievement. The light reflected by a sample of the objects used in our study changes noticeably under daylight and the four other illuminants we employed. In each case, the reflected wavelength composition and resulting cone excitations are very different. When viewed as isolated patches rather than full objects, the patches seem to change under the different illuminations, and we would frequently even assign a different color name to them. We investigated whether our participants are able to identify the same constant colors for these objects—purely from memory in the absence of the objects—under these different illumination conditions.

## Methods

### Participants

Sixteen subjects (five females) participated in this study. All subjects were members of the Department of Psychology of the University of Giessen and provided informed consent before taking part. Experiments were performed in agreement with the tenets of the Declaration of Helsinki and were approved by the local ethics committee (LEK 2013-0018). Twelve participants were naïve to the purpose of the experiment. Mean age of the participants was 35 years, with a range between 26 and 61. All observers had normal or corrected to normal visual acuity and normal color vision, as tested with the 24-plate edition of the Ishihara test for color deficiencies (Ishihara, 2018).

### Materials and methods

**Figure 2.**
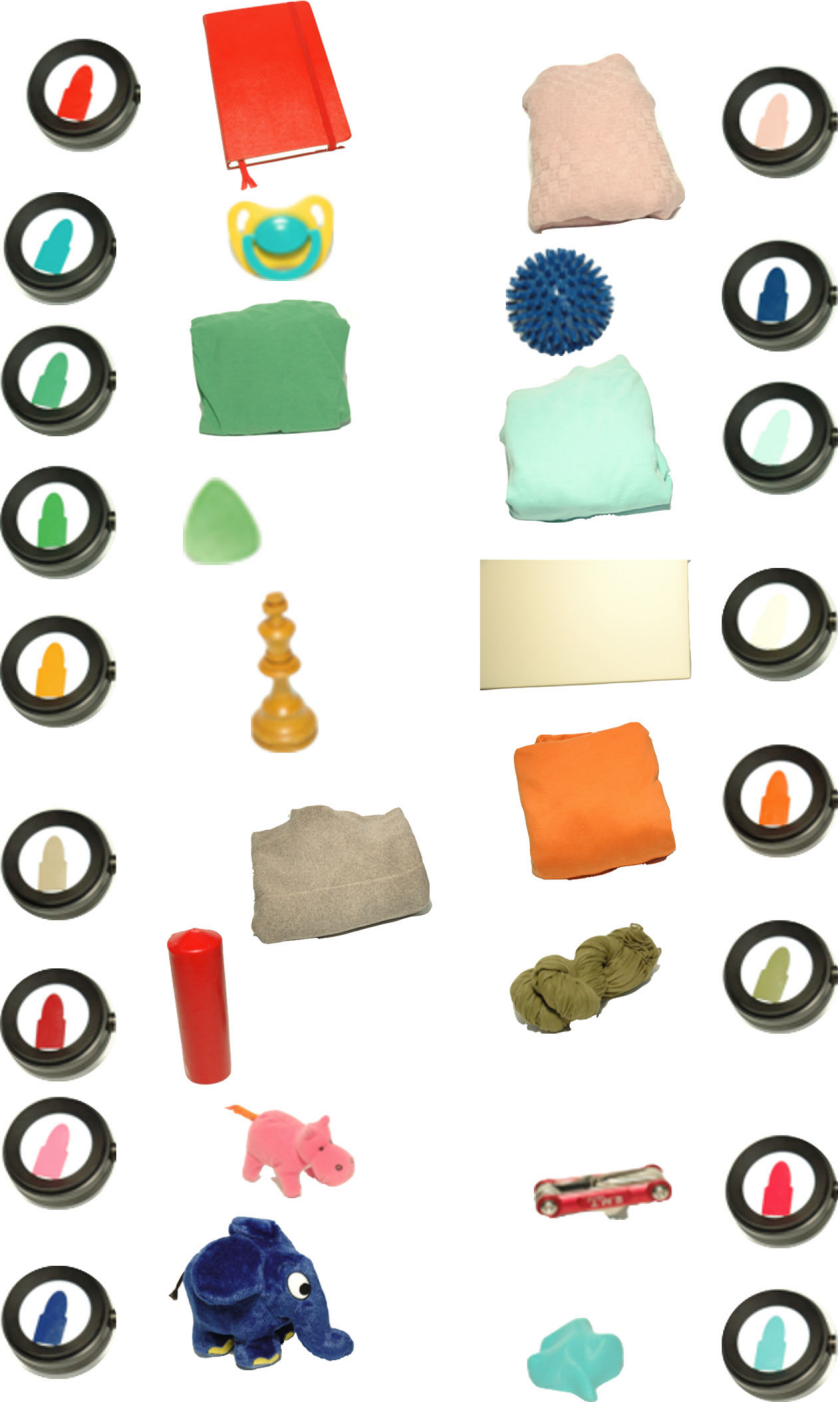
Photographs of the 17 objects brought by our participants. These photographs serve only to illustrate that each object indeed had a Munsell chip that matched its color quite well. To create these photographs, the experimenter selected the best-matching chip in the presence of the object. The photographs were in no way calibrated. Notice the variety of materials, sizes, and colors that represent the diversity of objects used in our study.

Subjects were asked to bring a personal object that was well known to them and that had a certain color that the participant was sure to have a good memory of. One subject provided two objects, a brown sweater and an orange sweater. Another subject only performed matches under two out of the four filter conditions for the blue spike massage ball. Photographs of the 17 objects are shown in Figure 2. The objects were taken away by the experimenter and were not seen by the participants until the end of the experiments. They were not present during the experiments.

### Procedure

**Figure 3.**
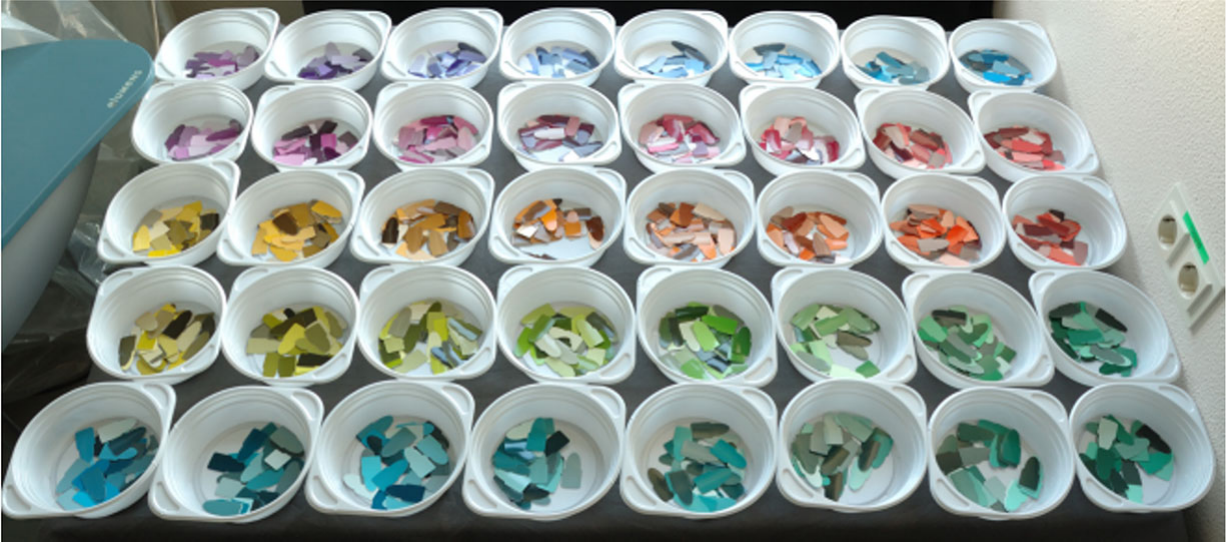
Photograph of the 1600 chips from the Munsell Glossy collection displayed in 40 plastic bins (here, under neutral daylight) as they were seen by observers after completing the adaptation task and before making their memory match selection.

**Figure 4.**
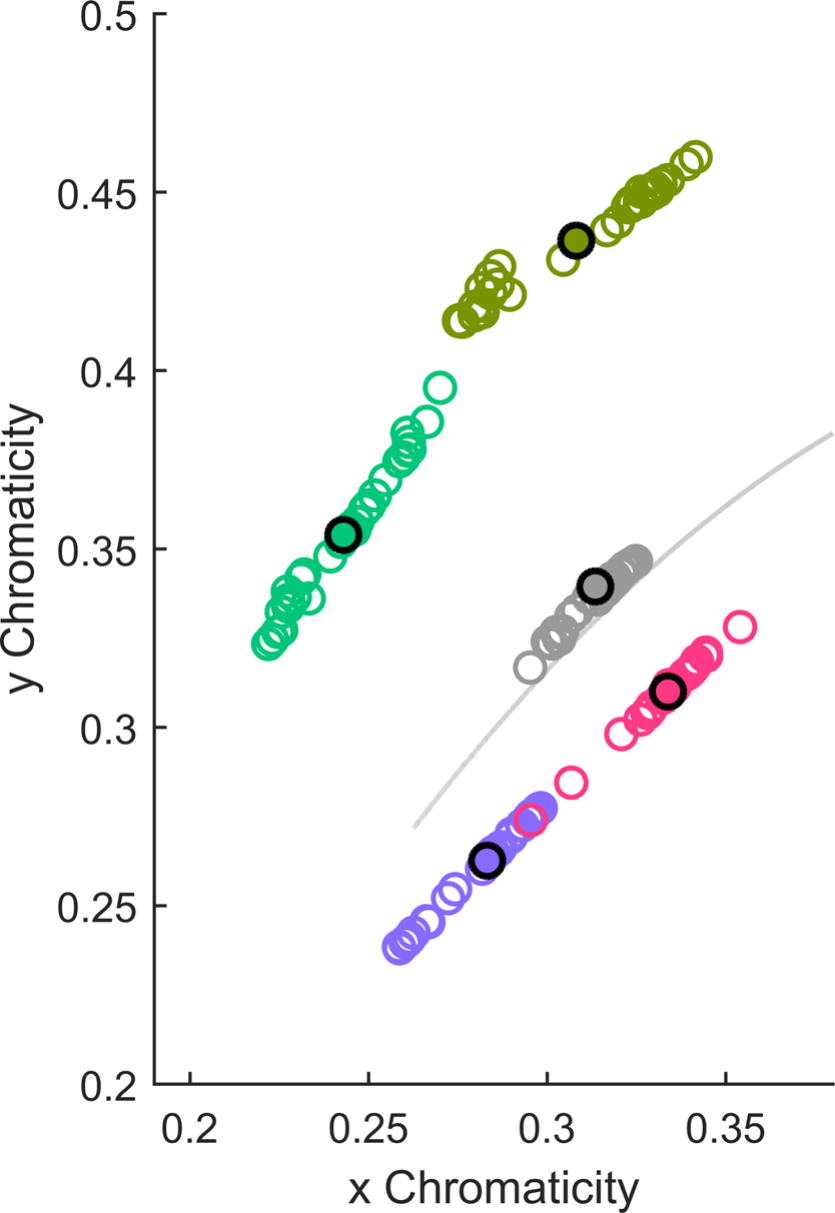
Judd–Vos-corrected CIE *x,y* chromaticities of the five illuminants measured in each session. Each colored circle represents the average of measurements before and after each session. The solid disk with a black outline represents the chromaticity of one illuminant averaged over all measurements. The solid gray line represents the daylight locus.

In the absence of their object, subjects were asked to select a chip from the *Munsell Book of Color (Glossy Finish Collection)* that best matched their recollection of the color of the object. The 1600 chips were arranged in 40 plastic bins by hue, as shown in Figure 3. Observers performed the chip selection task in two rooms of similar size, window aperture, and orientation to the sun path. Both had white painted walls and gray floors and were filled with experimental equipment, office furniture, and objects. The rooms were illuminated only by neutral daylight or by daylight modified by the use of one of four filters obtained from Lee Filters (Andover Corporation, Surrey, UK). The filters were attached in an inconspicuous way to the window in the rooms. Data for the neutral daylight and purplish filters were collected in the first room, data for the remaining three filters were collected in the other room. Details of the filters used and their chromaticity are provided in Table 1, Figure 4, and Table A1.

Before doing the chip selection, participants adapted for at least 2 minutes to the illuminant. The chips were arranged in bins, each containing one Munsell hue category of about 33 chips. The initial arrangement of the bins was in random order. During adaptation time, participants had to sort the bins by hue, so that the arrangement at the start of the selection task was as shown in Figure 3. This task allowed participants not only to adapt to the illuminant but also to become familiar with the Munsell chips. To keep participants from remembering the spatial position of the bin they chose a chip from in a previous trial, the absolute position of the bins was rotated after every session. A session consisted of two memory matches under a single illumination condition. Each participant ran five sessions, one under neutral daylight and one for each filter. The sessions were spaced over several weeks, during which the filters were changed on the windows. The observers did not see their object during that time.

Before and after each participant completed their selection procedure, the ambient illumination was measured by using a RS-2 (51-mm diameter) polytetrafluoroethylene (PTFE) reflectance standard (Photo Research, Syracuse, NY) and a CS-2000 Spectroradiometer (Konica Minolta Sensing, Singapore). The measured illuminants are shown in Table 1 and Figure 4. The variation within each illuminant is due to the different times of day the individual experiments were run. Figure 4 shows that neither of the filtered illuminants was aligned with the daylight axis. However, the differences between the yellow and green illuminants and between the red and violet illuminants closely parallel the daylight axis, whereas the differences between the yellow and red illuminants and between the green and violet illuminants are approximately orthogonal to the daylight axis.

### Data analysis

The most straightforward way to analyze our data is in the domain of the Munsell chips. An observer who is perfectly color constant would always select the same chip, irrespective of the particular illuminant. One way to quantify constancy is therefore to determine the proportion of such “perfect matches.” This is what we refer to as “Top1-accuracy.” The catalog of Munsell chips was empirically designed to cover all colors fairly evenly, so that neighboring chips would be perceptually approximately equidistant. If two chips that differ only in hue, chroma, or value are placed next to each other, they appear similar, but they can be well discriminated. However, if memory is involved, this is no longer the case and confusion occurs (e.g., Godlove, 1951). To account for these small inaccuracies, we also define a “TopN-accuracy,” which counts how often a chip was selected that was identical to the perfect one, or one of the 26 chips neighboring the perfect one in any of the three dimensions of the Munsell catalog. For hue, the chips fall into 40 different categories, as illustrated in Figure 3. There are 10 value categories. Chroma labels range from 2 to 16, but their spacing is 2. The probability of selecting the Top1 Munsell chip by chance is less than 1 in 1000, and that of selecting a TopN Munsell chip less than 17 in 1000.

For a more continuous analysis of errors, we chose to plot the chromaticity of selected chips in the approximately perceptually uniform color space CIE_1976_ L*a*b*. For this we used measured reflectance of the selected Munsell chips provided by the University of Joensuu Spectral Color Research Group (Orava, 2002) and average ambient illumination measured in our experimental rooms to calculate corresponding CIE XYZ values using Judd–Vos-corrected color matching functions (Judd, 1951; Vos, 1978), provided by the Color and Vision Research Laboratory (CVRL) database (http://cvrl.ucl.ac.uk/), and converted them to CIE _1976_ L*a*b* (Wyszecki & Stiles, 2000).

For some object/illumination combinations, the *chromaticity* of the chip selected by the owner under daylight was no longer represented in the Munsell collection under a different illuminant. For example, under the greenish illumination, there is no Munsell chip that matches the chromaticity coordinates of the “red book” object under the neutral illumination. Thus, zero color constancy cannot be obtained under these conditions, because any selected chip will be shifted toward the “red book” object under the greenish illuminant (i.e., will tend toward constancy). In these cases, we calculated for the corresponding object/illumination combination the lower bound of color constancy using the chromaticity of the chip that under that illuminant would provide the lowest possible constancy index. The calculated lower bounds for constancy are shown in the bar charts later on as dark shaded regions. The problem emerges only for a few object/illumination combinations and is not systematically related to the overall high level of constancy we obtained.

### Metamer analysis

To calculate potential metamers of the Munsell chips selected by participants in our study as memory matches under neutral illumination, we compared the spectral distribution of each of them to 11,302 surface spectra gathered from different online databases (Arnold, Savolainen, & Chittka, 2008; Barnard, Martin, Funt, & Coath, 2002; Berns, 2017; Haanpalo, 2017; Hiltunen, 2017; Jaaskelainen, Silvennoinen, Hiltunen, & Parkkinen, 1994; Marszalec, 2017; Matsumoto et al., 2014; Orava, 2002; Parkkinen, Jaakelainen, & Kuittinen, 1988; Regan et al., 1998; Westland, Shaw, & Owens, 2000), leading to 192,117 (17 × 11, 302 – 317) possible comparisons (for details, see Akbarinia & Gegenfurtner, 2018). Each reflectance was rendered under each of the five illuminant spectra used in our experiment. The resulting spectral product was converted to CIE_1931_ XYZ values using CIE_1931_ 2°-observer color matching functions. To make the illuminants comparable for this analysis, the luminance according to an ideal reflector was normalized to *Y* = 1 before calculating the spectral product. Further, to be able to examine perceived differences between two surface reflectances, XYZ tristimulus values were converted to CIE L*a*b using the white point of the given illumination. Metamer pairs were defined using a threshold limit in perceived differences (CIE ∆*E*2000) (Luo, Cui, & Rigg, 2001) in CIE L*a*b. If two surfaces were below that threshold under one illumination and above it under another illumination, they were regarded as metamers. There is no official agreement on how CIE ∆*E*2000 is related to just notable differences (JNDs). The MacAdam ellipses range in CIE ∆*E*2000 values from 0.23 to 0.89, with a mean of 0.5. We chose a slightly higher threshold of CIE ∆*E*2000 = 1.5 for defining metameric matches.

We also calculated metameric mismatch volumes using the approach suggested by Logvinenko, Funt, and Godau (2014), as described by Witzel et al. (2016). For each reflectance of the chips selected by participants under neutral daylight, metameric mismatch volumes were calculated for the four illuminant changes from neutral daylight to the chromatic illumination in CIE L*a*b.

## Results

As laid out in the Methods above, our analysis is twofold. We first present the results solely as the discrete selections of our observers in the Munsell chips catalog. We then map these selections into the CIELAB color space and analyze them in a more continuous manner.

### Discrete analysis in munsell space

**Figure 5.**
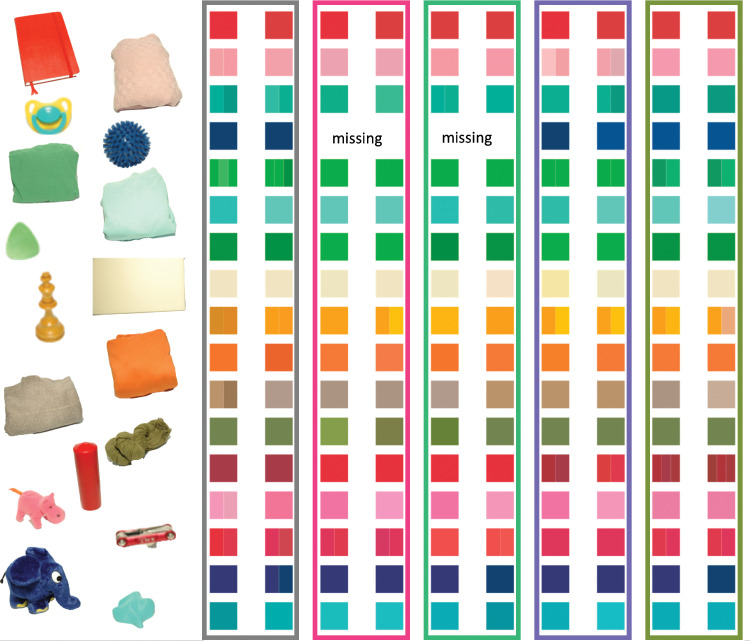
Rendition of the Munsell chip selections of all 17 observers under the neutral (gray bounding box) and the four chromatic illuminants. The two columns per illuminant show the two repeats. Sometimes, more than one color is rendered within a single square. That is the case when observers were uncertain and selected more than a single Munsell chip as their match (see also Table A2). For rendering, the CIE XYZ values provided by the University of Joensuu Spectral Color Research Group (Orava, 2002) chips were converted to standard RGB (sRGB).

Figure 5 shows an RGB rendering of the selections, purely for illustration purposes. Each row is for one of the objects. The 10 colored patches show the selections made for the five illuminations and the two repeats in each session. In some cases, individual patches are subdivided in the rare cases when an observer selected more than a single chip. Even though the illustration cannot reproduce the appearance of the individual chips in a totally accurate manner, it does convey the main result of our work quite well. The differences between the chips in each row are relatively modest, meaning that observers’ matches are highly consistent across illuminations, achieving a high degree of constancy. All observer choices in standard Munsell notation are listed in Table A2.

**Figure 6.**
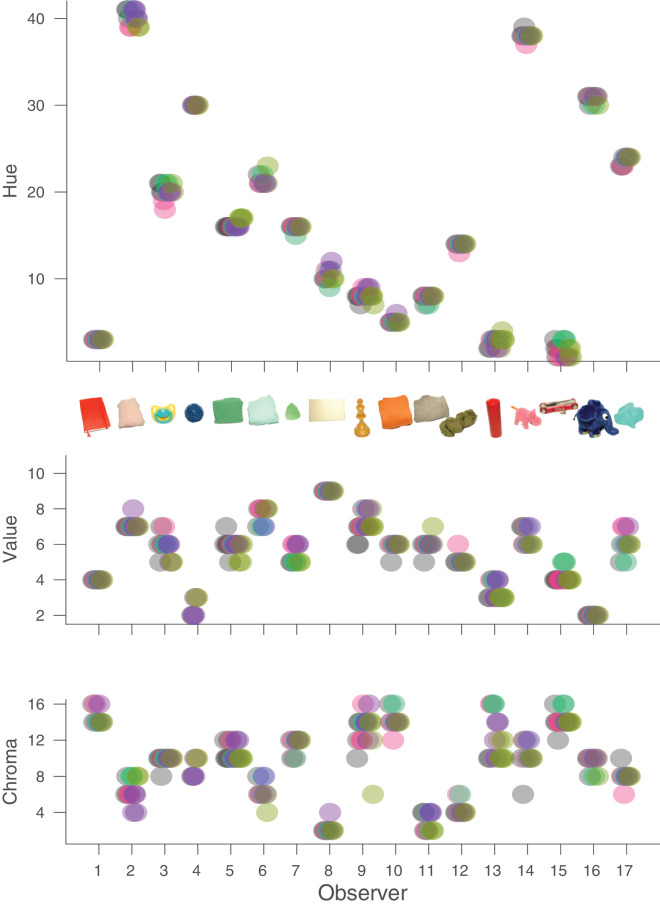
Distribution of the observers’ selections of Munsell chips. For each observer, we plotted Munsell hue (top), (Middle) Munsell value (middle), and Munsell chroma (bottom) of all selected chips. The different colors indicate the five different illuminants. Small horizontal offsets were introduced to make all of the data points visible. Some observers have more than 10 data points because they kept several choices for particular conditions (see also Figure 5 and Table A2).

Figure 6 shows the same selections of our observers with respect to Munsell hue, value, and chroma. If observers were perfectly color constant, there would only be a single point visible above the x-axis for each observer. Because data points were indeed frequently overlapping for individual observers, we added small horizontal offsets to retain visibility. Figure 6 indicates that there was hardly any deviation for Munsell hue and relatively little deviation with respect to value and chroma. In the following, we regard the selections of the observers under the neutral illumination as the “correct” one and consider the deviations in a more quantitative manner. We define *bias* as the average signed deviation from the correct choice. *Accuracy* (or inaccuracy, to be accurate) is defined as the average absolute deviation from the correct choice. *Precision* is defined as the standard deviation of the signed deviations from the average choice. In the following, we also average multiple choices under each condition and average across the two repeats, so that we are left with five settings for each observer (or three for observer AZ, who completed only two illumination conditions).

**Figure 7.**
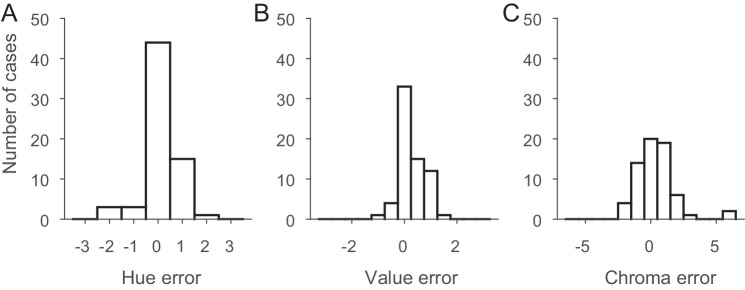
Histograms of the differences between the selections under the neutral illuminant and for the colored illuminants for all objects. Distributions are shown for Munsell hue (**A**), Munsell value (**B**), and Munsell chroma (**C**).

**Figure 8.**
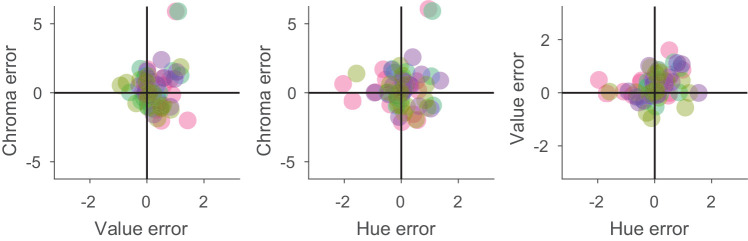
Two-dimensional distribution of selection errors in Munsell space. The different colors indicate the color of the four different illuminants. Small random offsets were introduced to make overlapping data points visible.

In Figure 7, we have plotted histograms of these deviations. In all three panels, the *x*-axis covers three neighboring chips on each side of the Munsell catalog. Note that the range for chroma is larger because Munsell chroma increases by steps of two for all but extremely unsaturated chips. Figure 8 shows the two-dimensional distribution of these errors.

Figures 6, 7, and 8 reveal that the selections made by our observers were highly accurate. In 35% of all cases, they chose the correct hue, and in 95% of all cases they chose the correct one or the neighboring one. Only in four cases did observers on average choose a hue that deviated by more than one Munsell step, and in all of these cases the deviation was less than or equal to two steps. Because hue is not a directional property, we would not expect any bias for hue, and indeed we did not observe one, bias = 0.037, *t*(65) = 0.46, *p* = 0.65. Accuracy, the average absolute deviation from the correct choice (the selection under the neutral illumination), was 0.43, and well within one Munsell step. Precision, the standard deviation of the signed deviations from the average choice, was 0.65, also less than one Munsell hue unit.

The choices with respect to Munsell value were similarly accurate (accuracy = 0.35) and precise (precision = 0.47). However, value was the only dimension with a significant bias of 0.245, *t*(65) = 4.21, *p* < 0.0001, indicating a tendency for observers to choose a Munsell chip with a slightly higher reflectance than their neutral choice. This was not correlated with the other choices and did not depend on illumination. Observers chose the correct Munsell value in 45% of cases, and, if they did not pick the correct one, they always picked the one just below or just above the correct one.

Observers picked the correct chroma in 23% of all cases, and in 97% of all cases they were no more than one chip apart from the correct choice. The only two larger errors were for the red candle under the reddish and greenish illuminants, where the chroma was highly overestimated (chroma of 16 rather than 10). Overall, the bias for chroma (0.247) was not significant, *t*(65) = 1.37, *p* = 0.17. Accuracy was 0.96 and precision was 1.44. Although these numbers are higher than for value, one has to keep in mind that the difference in chroma between two neighboring Munsell chips is two, rather than one for hue and value.

When considering the selections in all three dimensions at the same time, we found a Top1-accuracy of 50%. That is, about half of the time, observers picked the same Munsell chip under the four experimental illuminants that they had selected under the neutral illuminant. TopN-accuracy was 90%. In nine out of 10 trials, observers selected either the perfect or a neighboring chip. This reflects quite a remarkable degree of constancy that is actually close to the limits of the observers’ memory for these objects (Bloj, Weiss, & Gegenfurtner, 2016). In fact, we did find that the light brown chess piece under the yellowish illuminant led to particularly poor constancy. In our previous study on memory color for the objects used in this experiment (Bloj et al., 2016), we also found the highest variance in chroma for the chess piece compared with all other objects even in object-present matches, so we suggest that the object itself is difficult to match due to its surface properties.

**Figure 9.**
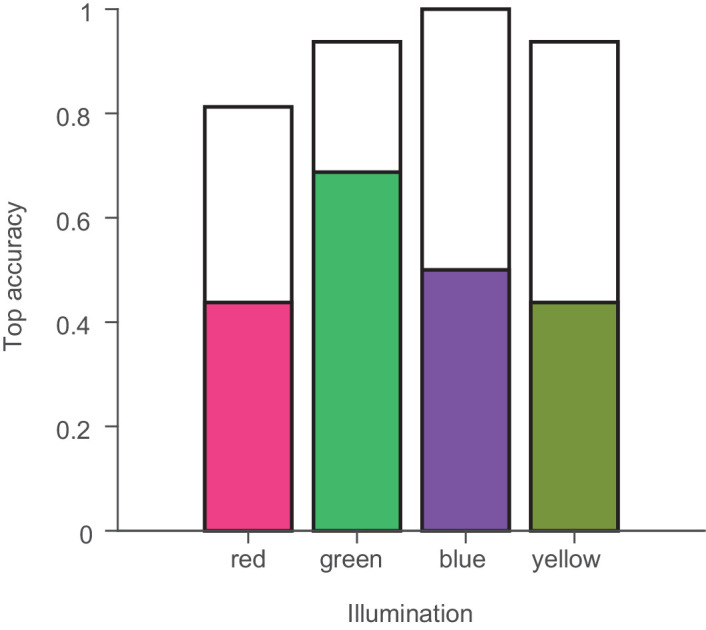
Top accuracies separately for the four different experimental illuminants. The filled bars indicate the proportion of cases in which the exact matching Munsell chip was chosen under the four experimental illuminants by the color of the bars. The open bars indicate how often an immediately neighboring chip was chosen.

So far, we have considered all illuminations as being equal. One long-standing question in color constancy research has been whether there are differences in the degree of constancy for different illumination conditions. In particular, the idea that the visual system has adapted to the type of illumination changes occurring naturally has been often tested with, so far, inconclusive results (Delahunt & Brainard, 2004; Hedrich, Bloj, & Ruppertsberg, 2009; but see Pearce, Crichton, Mackiewicz, Finlayson, & Hurlbert, 2014). Figure 9 shows the accuracies for each illuminant separately. There were only unsystematic differences between the Top1-accuracies and TopN-accuracies under the different illuminants, *F*(3, 45) = 1.13, *p* = 0.34. However, none of the illuminations we considered fell on the potentially privileged daylight axis in color space. But, our choice of illuminations does give us a way to analyze the results with respect to this question.

**Figure 10.**
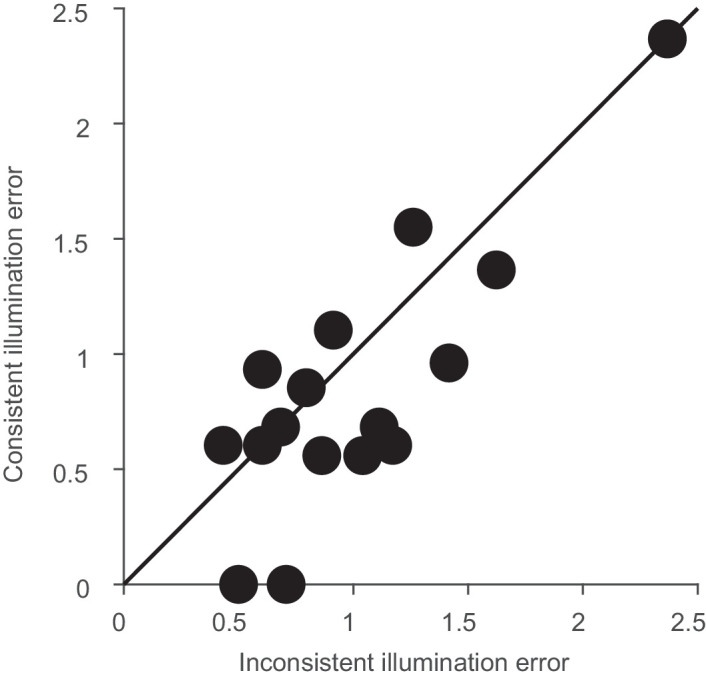
Selection errors as distances in Munsell space. Error was calculated as the Euclidean distance between hue, value, and 0.5*chroma settings. A value of 1 indicates the distance between two neighboring Munsell chips. Consistent illumination changes were along the daylight variation indicated in Figure 4, whereas inconsistent changes were roughly orthogonal.

Figure 4 shows that the illumination variations we observed during the day closely matched the difference between the yellowish and greenish illuminants and between the reddish and bluish illuminants. If we are less sensitive to daylight variations, then the matches might agree better between these two sets of illuminants, which we refer to as “consistent.” The other two pairings, between the alternative two sets, greenish and bluish versus yellowish and reddish, we refer to as “inconsistent,” because they differ along an axis roughly orthogonal to the daylight axis. We therefore calculated the Munsell space difference between these pairwise settings for each observer. The averaged errors in Munsell space (hue, value, 0.5*chroma) are plotted in Figure 10. Errors are significantly smaller for the illumination changes consistent with daylight changes than for the inconsistent changes, *t*(15) = 2.43, *p* < 0.05.

Taken together, our analysis of the observers’ selections in Munsell color space indicates a rather high level of color constancy in our natural environment. In the following, we present a second analysis, where we transform our data into CIELAB space. This allows us to calculate measures of color constancy that are better comparable to previous studies.

### Continuous analysis in CIELAB space

**Figure 11.**
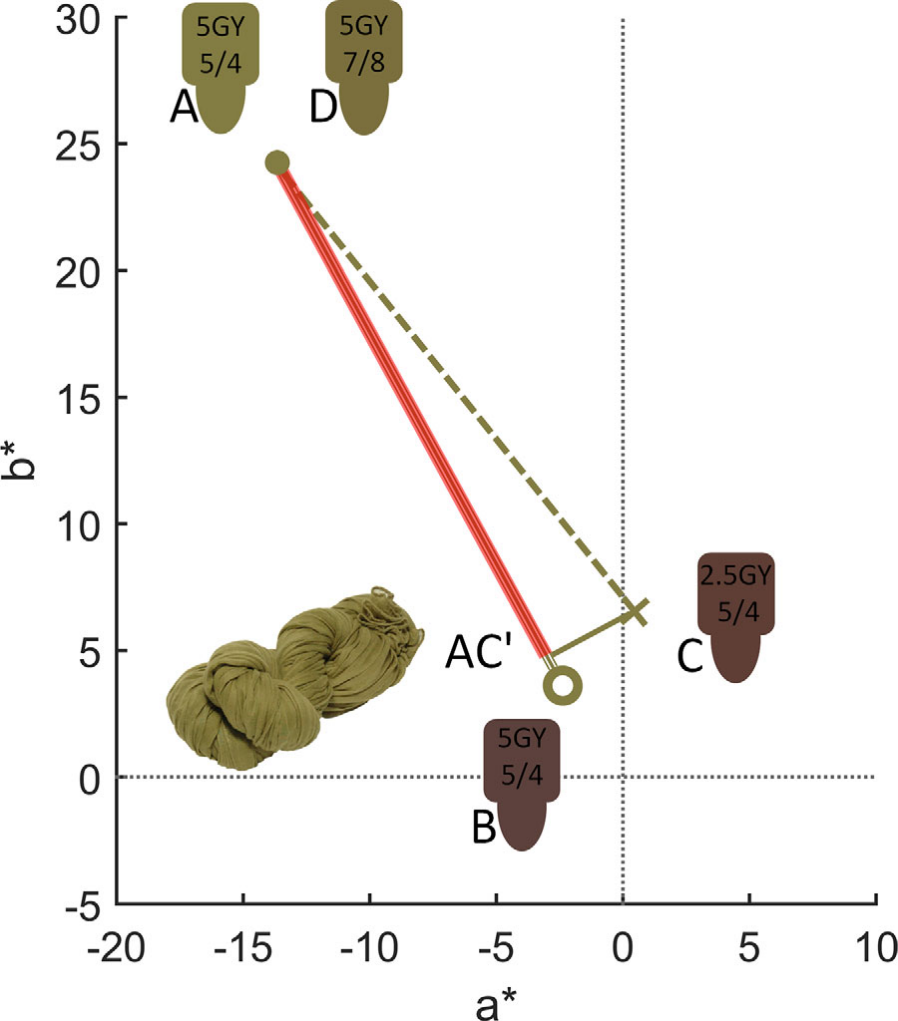
Representation in CIE a*b* plane of the color coordinates of the chip selected by the owner of the green scarf under neutral daylight illuminant (A, dot), the coordinates of that same chip under one of the test illuminants (B, circle), and of an alternative chip under the same illuminant (C, cross). AC’, indicated by a red line, gives the vector projection of AC onto AB. D represents a zero constancy choice for this object-illuminant combination.

Figure 11 illustrates the basic results in CIELAB color space using the green scarf as an example. For this object, under neutral daylight illuminant_,_ the owner selected chip 5GY5/4 (represented by point A in Figure 11) as a match to their object. The matches, as well as those under the other illuminants, were made from memory, as the actual object was not present. If this participant were fully color constant, then they would also select chip 5GY5/4 under all four experimental illuminants (represented here, under the purplish illuminant by point B in Figure 11). If they were less constant they might select an alternative chip such as 2.5GY5/4 (represented by point C in Figure 2). We computed the corresponding color constancy index (CCI) by projecting vector AC onto AB (indicated by AC’). When C and B are identical, the index equals 1 (expressed as 100%), indicating perfect color constancy. Zero constancy would instead be represented if the observer would pick a chip that has an equal color signal under the purplish illuminant as the memory match under neutral daylight, in this case 5GY7/8 (represented by point D in Figure 11). Note that this index, usually referred to as Brunswick ratio (BR; see Foster, 2011) can achieve values larger than 1. We discuss advantages and disadvantages of various indices later on. The main advantage of the BR over other measures is that it is an unbiased estimator in the presence of noise. If the true value of color constancy is 1, then the BR will estimate it to be 1, whereas other indices might underestimate constancy.

**Figure 12.**
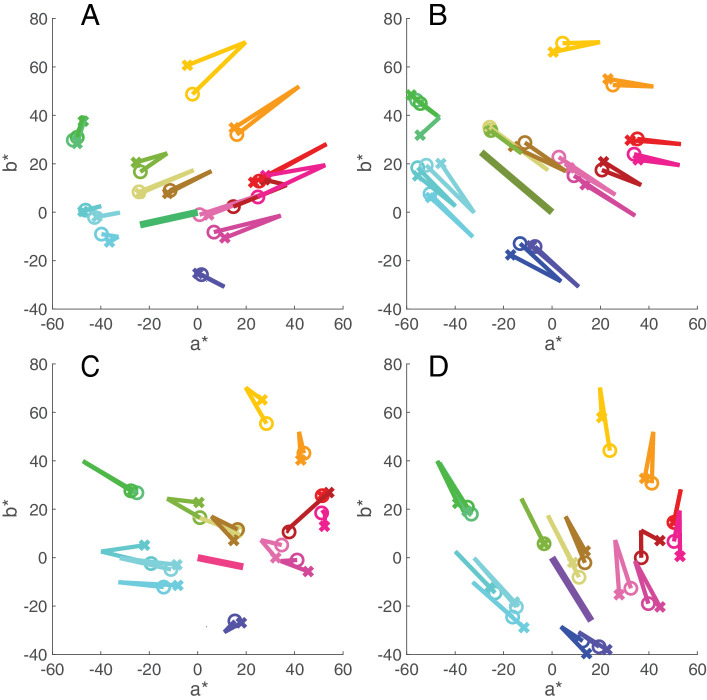
Diagrams showing in CIE a*b* plane the direction and magnitude of the four illuminant shifts (**A**, greenish; **B**, yellowish; **C**, reddish; **D**, bluish) with a heavy line that starts at the chromaticity of neutral daylight and ends at the chromaticity of the test illumination. As in Figure 11, the lines for each object start at the chromaticity of their memory match under neutral daylight and extend to their memory match under the new illuminant, represented by crosses. The line ending in a circle represents the chromaticity shift of the memory selection for a given object from neutral daylight to the test illuminant. Plots are averages over two sessions. Changes in the lightness plane are not shown here.

**Figure 13.**
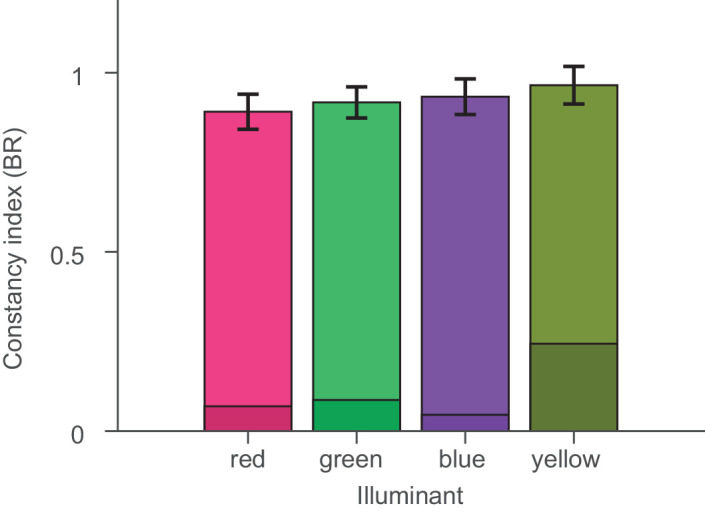
Constancy indices averaged over all objects for each test illuminant. Shaded regions indicate lowers bounds for color constancy as described in Methods.

**Figure 14.**
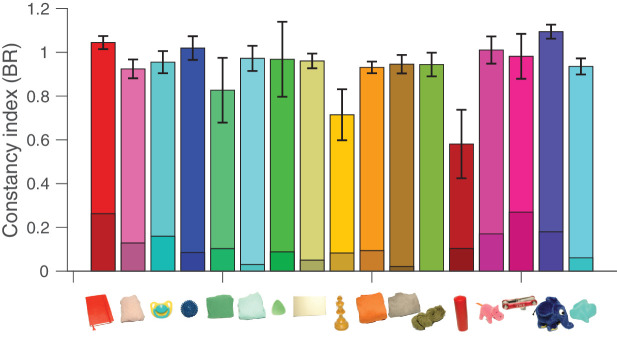
Color constancy indices averaged over all illuminant conditions for each object. A value of 1 indicates perfect color constancy, and error bars represent standard errors of the mean. Shaded regions indicate lower bounds for color constancy as described in Methods.

Figure 12 shows the results for all objects under the four experimental illuminants, averaged over two sessions. For each object, two lines emerge from the memory color selection under daylight. One line connects the color coordinates of the selected chip under daylight to the color coordinates of the same chip under the new illuminant (represented by a circle). The second line extends to the color coordinate of the chip the observer selected from memory under the new illuminant (represented by a cross). Figure 12 shows that for most objects these lines stay close together and frequently overlap. Constancy is close to perfect under all conditions, with a mean value of 93.9% (±20% *SD*) and a median of 99.2% across all objects and illuminants. There was small variation between illuminants, as shown in Figure 13, and some variation between objects (and observers), as shown in Figure 14.

**Figure 15.**
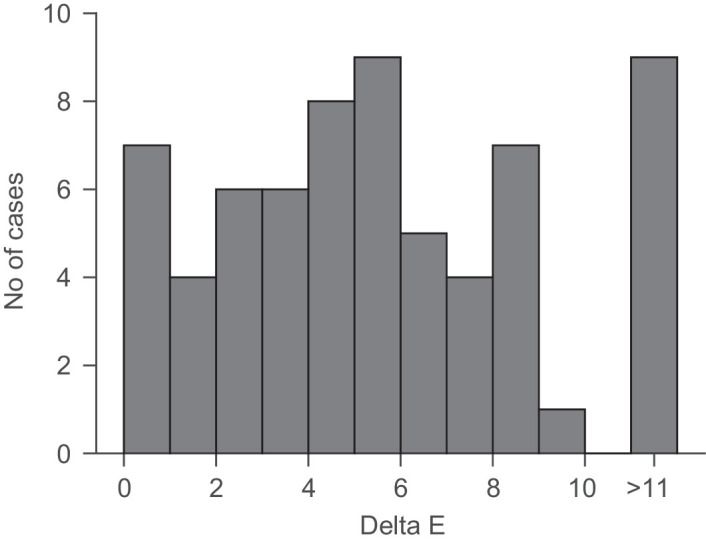
The bars depict the frequency of deviations from a perfect color constant match in Euclidian distances in CIE L*a*b* color space. Multiple selections and the two repeats (see Table A2) were averaged before calculating ∆*E*. The total number of matches was therefore 66. The trough in the distribution at small values is expected due to the discrete nature of the Munsell chip system.

Another way to evaluate constancy is to look at the differences between the perfect match and the selected chip. In the CIE_1976_ L*a*b* color space, the Euclidean distance ∆*E* to a first approximation represents a perceptual JND, based on measurements by MacAdam (see Brainard, 2003). From a previous memory study (Bloj et al., 2016), we know that the reliability of visual long-term memory for our participants and objects is of the order of one Munsell step in hue, one and a half in chroma and half a step in value (see Figures 3 and 4 of Bloj et al., 2016). This roughly corresponds to the distance between two neighboring Munsell chips, which is roughly equivalent to 5 ∆*E* units for the 17 chips selected under neutral daylight in this study. The observed deviations between the selected chip and the chip representing perfect color constancy were small and of the same order as this memory limit (mean ∆*E*, 5.5; median ∆*E*, 5.14 ± 3.5 *SD*, ±0.43 *SE*), as shown in Figure 15 (see also Table 2). In 23 out of 132 cases, participants even selected the very same chip under the test illuminant as under natural daylight. The bimodal shape of the histogram arises due to the discreteness of the collection of chips. This means that our observers, with just a few exceptions, were as good as would be expected based on their visual memory.

### Analysis of metamers

It has been argued many times that, in theory, color constancy is impossible to achieve because of the metamer mismatch problem (e.g., Foster, Amano, Nascimento, & Foster, 2006; Logvinenko et al., 2015). The spectral distribution of light reflected from an object varies continuously in wavelength. In the eye, it gets reduced to three numbers: the excitation of long-, middle-, and short-wavelength-sensitive cones (Stockman & Sharpe, 2000). Consequently, there must be many physically different spectra that lead to the same cone excitations under a particular illumination but which may differ under another illumination. In practice, the volume of color space under one illumination that gets mapped into a single point under a second illumination has been shown to be quite large, and a recent study suggested that the size of that volume is negatively correlated to some measures of color constancy (Witzel et al., 2016). The open question is whether the metamers actually occurring in nature do present a problem for achieving color constancy (Akbarinia & Gegenfurtner, 2018; Foster & Reeves, 2022; Zhang, Funt, & Mirzaei, 2016). We therefore computed the size of the metameric mismatch volume for the colors of the objects used in our^1^,^2^ study (see Table 3). If the magnitude of the mismatch volume determines the degree of constancy, then there should be a correlation between these two quantities. A larger metameric mismatch volume would go along with poorer color constancy. There was no significant correlation across objects with the CCI, with all *r*(15) between –0.32 and 0.24 and all *p* > 0.21 (Table 4). Also, the average mismatch volume was smallest for the reddish illuminant, which then should also have the best color constancy. This was not the case, as constancy was worst under the reddish illuminant.

**Table 1.** Illuminant specifications and averaged Judd–Vos corrected CIE *x*,*y*, and *Y* coordinates of the white standard measurements. The values are means (*SD*) over all sessions for a specific illuminant. See also Table A1.

Illuminant	Lee filter specification	*x* mean (*SD*)	*y* mean (*SD*)	*Y* (cd/m^2^) mean (*SD*)
Daylight	—	0.3135 (0.0103)	0.3395 (0.0159)	186 (63)
Green (A)	242 Fluorescent 4300K	0.2430 (0.0148)	0.3539 (0.0203)	80 (33)
Yellow (B)	138 Pale Green	0.3082 (0.0234)	0.4364 (0.0155)	175 (88)
Red (C)	035 Light Pink	0.3338 (0.0109)	0.3100 (0.0103)	67 (47)
Violet (D)	136 Pale Lavender	0.2832 (0.0143)	0.2627 (0.0143)	84 (31)

**Table 2.** Values of ∆*E* under each illuminant change from neutral to chromatic illumination in CIE L*a*b. Multiple selections and the two repeats (see Table A2) were averaged before calculating ∆*E*.

Object	Reddish	Greenish	Blueish	Yellowish
Red book	0.00	2.36	0.00	3.01
Light pink scarf	5.81	3.80	5.80	6.25
Turquoise dummy	8.59	1.31	4.12	8.72
Blue spike massage ball			6.25	9.00
Green t-shirt	1.95	2.07	2.23	14.66
Turquoise sweater	4.47	3.77	2.20	7.01
Green pick	7.55	7.12	8.37	5.19
Light yellow part of kitchen	0.61	0.77	6.65	0.00
Wooden chess piece	11.66	13.71	15.79	8.22
Orange sweater	4.36	4.46	4.91	5.36
Brown sweater	5.10	3.10	5.17	8.46
Green scarf	7.12	4.31	0.00	0.00
Red candle	24.21	17.55	11.05	3.52
Pink hippo	6.64	5.36	5.10	8.34
Red metallic bike tool	5.63	11.26	6.66	2.87
Blue elephant	2.76	1.58	3.45	1.55
Turquoise potato	11.63	4.63	9.24	4.32

**Table 3.** Size of metameric mismatch volumes under each illuminant change from neutral to chromatic illumination in CIE L*a*b.

Object	Reddish	Greenish	Bluish	Yellowish
Red book	91.191	272.113	130.843	226.491
Light pink scarf	171.448	571.134	294.767	476.592
Turquoise dummy	161.747	803.340	338.545	561.796
Blue spike massage ball	102.432	626.760	165.480	269.364
Green t-shirt	240.930	816.007	350.736	804.361
Turquoise sweater	108.465	311.016	145.092	268.112
Green pick	246.439	487.150	247.779	737.481
Light yellow part of kitchen	71.585	263.121	135.139	223.991
Wooden chess piece	169.322	269.004	145.645	399.401
Orange sweater	154.386	329.545	161.807	343.679
Brown sweater	260.950	771.170	361.586	750.768
Green scarf	297.946	593.253	306.530	758.085
Red candle	92.978	276.204	140.267	233.110
Pink hippo	154.255	517.018	232.771	327.631
Red metallic bike tool	84.250	313.942	145.533	203.338
Blue elephant	112.151	640.097	184.789	267.858
Turquoise potato	187.935	815.161	365.093	580.422
Average volume	159.318	510.355	226.612	437.205
Cube root	5.421	7.991	6.097	7.590

**Table 4.** Correlations across all objects between metameric mismatch volumes and color constancy for each illuminant change. MMV, metameric mismatch volume; BR, Brunswick ratio; CCI, color constancy index.

	Illuminant change			
Illuminant change	Reddish	Greenish	Bluish	Yellowish
MMV and BR correlation	−0.060	0.240	−0.130	−0.320
*p*	0.820	0.350	0.610	0.210
MMV and CCI correlation	0.21	0.20	0.31	−0.38
*p*	0.42	0.46	0.21	0.13

We also computed the frequency of metamers to the selected Munsell chips within a large set of 11,302 natural reflectance spectra (Akbarinia & Gegenfurtner, 2018). Metamers were infrequent (5.1 × 10^−04^) for the illumination changes used in this experiment. On average, there were 5.76 ± 10.32 (see Table 5) metamers per object, but only the green scarf (42 metamers) and the red book (16 metamers) had a larger number of metamers. For eight objects, there was no metameric surface within the whole set of natural reflectance functions. There was no significant correlation between the number of metamers and the degree of color constancy (rho = 0.21, *p* = 0.43). This held for CCI and BR and also for several different combinations of ∆*E* criteria to calculate the metamers. Based on these calculations, we do not think that metameric mismatch represents a big problem for color constancy under these experimental conditions.

**Table 5.** Number of metamers for the objects tested in our study. Frequencies are given for the five illuminants used in our experiment, out of 11,302 reflectance samples.

Object	Number of metamers
Red book	16
Light pink scarf	6
Turqouise dummy	0
Blue spike massage ball	8
Green t-shirt	0
Turqouise sweater	0
Green pick	0
Light yellow part of kitchen	0
Wooden chess piece	4
Orange sweater	4
Brown sweater	4
Green scarf	42
Red candle	0
Pink hippo	6
Red metallic bike tool	0
Blue elephant	8
Turquoise potato	0

## Discussion

Color constancy, in particular the degree to which it holds, has been a topic of active debate for over a century. Experimental findings on color constancy vary widely, with reports of it being as low as 15% to as high as 80%. This range seems at odds with the generally stable appearance of colors in the real world and the uniform way we name colors. Measurements of color naming under different illuminations (Olkkonen et al., 2009; Olkkonen et al., 2010) have shown that 40% to 50% of the Munsell chips from the World Color Survey (Berlin & Kay, 1991) would change to another color category without constancy. In our study, we have demonstrated color constancy approaching perfection using naturalistic settings more typical for daily life. The most important differences between our study and most others are (1) the use of an individual long term memory stimulus as the test object, (2) a dense sampling of color space by using a selection paradigm with more than 1000 Munsell chips, and (3) the use of the BR as an unbiased estimate of the degree of constancy. In the following, we discuss these features individually and relate them to the known mechanisms of color constancy.

### Tasks

If we want to measure the degree of color constancy, color appearance must be measured under at least two different illuminations. This implies that some form of matching has to be performed either across space or across time. The most straightforward and efficient way to achieve such matches is to have different illuminants in different parts of the visual field.

In such classic experiments using simultaneous asymmetric matching, participants are then asked to adjust the color of a match object or surface seen under one of the illuminants to the color of the test object shown under the other illuminant. Color constancy in this paradigm turned out to be fairly poor, between 13% and 50%, especially when surfaces were simulated flat, matte patches displayed on small computer screens (Arend & Reeves, 1986; Arend, Reeves, Schirillo,& Goldstein, 1991; Tiplitz et al., 1988). Furthermore, the results depended on the instructions given to the observer. When the observer was asked to make the two patches under different illuminations *appear* the same, constancy was quite low. When the observer was asked to adjust the two patches as if they were *made from the same paper*, constancy was higher. In both cases, participants’ adaptation to either illuminant was compromised due to the constant presence of two illuminants, the relatively small region of the visual field illuminated by each illuminant, and by eye movements. Although there are examples of studies using asymmetric matching paradigms that achieved higher degrees of constancy (Bäuml, 1999; Troost & de Wert, 1991), on average these designs led to constancy values of about 50% (Foster, 2011). It should be noted that constancy in asymmetric matching can also be improved by using a more immersive lighting environment (Speigle & Brainard, 1999) or a more natural selection task (Radonjic et al., 2015a; Radonjic et al., 2015b).

Kraft and Brainard (1999) used an achromatic matching task (see Brainard, 1998) with a single illuminant to which participants were well adapted. Their work elegantly showed that constancy can vary between 20% and 80%, depending on the richness of the cues available to the observers. They found that three factors—global scene color, local contrast, and the color of the brightest region—had an effect on color constancy. Two problems remain, though. First, the use of this technique where participants are asked to adjust the patch until it appears gray is distinct from how we experience color in our daily lives. Thus, it cannot provide a general test because it is limited to the single point in color space void of any color. In our experiments, we used participants’ internal representations of many colors as shown by the good coverage of the color space in Figure 12 and the variety of objects in Figure 2. But, even when all cues are provided, constancy in the Kraft and Brainard (1999) study remained significantly below perfection. This may or may not indicate that color constancy is distinct from other constancies. For size constancy, for example, the availability of different cues also plays a big role. When distance cues are poor (for example, at large distances), size constancy can be quite poor (e.g., Kaufman & Kaufman, 2000). When sufficient cues are provided, constancy can be perfect (Holway & Boring, 1941).

A few studies have obtained even higher values of constancy. These studies have used either color naming (Troost & de Wert, 1991; Smithson & Zaidi, 2004) or color categorization (Hansen, Walter, & Gegenfurtner, 2007; Olkkonen et al., 2009; Olkkonen et al., 2010) to approximate the hypothesized function of color as an identity tag in our daily lives. Their results show that the appearance of objects under different illuminations typically does not cross into a different color category. Although both naming and categorization remain very much intact under different illuminants, they provide a relatively coarse sampling of our sensory and perceptual color space due to linguistic limitations. In our current study, we have shown that color constancy is better than just categorical. Of course, Munsell space is also not a continuous representation of the millions of colors we may be able to discriminate under ideal circumstances. It is very close, though, to the perceptual graininess of color space, which amounts to about 1000 different colors (Koenderink, van Doorn, & Gegenfurtner, 2018).

A crucial aspect of our work is, as often in real life, the use of memory to make the measurement of constancy for absent colored objects possible. In some previous work (Allred & Olkkonen, 2013; Hedrich et al., 2009; Ling & Hurlbert, 2008; Uchikawa, Kuriki, & Tone, 1998), memory performance was taken into account when calculating the degree of constancy. These authors found that, for real two-dimensional paper patches (Uchikawa et al., 1998; Ling & Hurlbert, 2008), real three-dimensional paper cubes (Allred & Olkkonen, 2013), or both (Hedrich et al., 2009), constancy was as good as memory allowed for. Our experiments differ in that we used objects that were already highly familiar to our participants (see also Olkkonen, Hansen, & Gegenfurtner, 2008; Smet, Zhai, Luo, & Hanselaer, 2017). We can exclude a possible role of memory biases (Bloj et al., 2016) in the present study by only comparing memory matches across different illuminations. Our results show that color constancy is indeed close to perfection when a natural task, natural illumination conditions, and natural stimuli are used in combination.

### Indices

Our results show that performance in a color constancy task can be close to perfect under ideal conditions. Previous studies have often used more impoverished paradigms to get performance into a range where the effect of different cues could be investigated. Still, in all of these studies, a ceiling on the very best performance emerged at levels of about 80% constancy. Our results prove that no such hard ceiling exists. One aspect that may have led to sometimes relatively low estimates for the degree of constancy concerns the estimates themselves (for review, see Foster, 2011). The frequently used CCI, dividing the difference between adjusted color and perfect match by the magnitude of the overall colorimetric color change, is only an unbiased estimate of constancy in the absence of noise. This seems unrealistic, as some degree of noise is even present under highly optimized conditions used to measure color discrimination thresholds (Krauskopf & Gegenfurtner, 1992; MacAdam, 1942). Foster et al. (2001) showed that such noise can be reduced by averaging the matches across many observers. In their case, this led to an improvement of the CCI from 60% to 70%. When memory becomes involved in such matches, adjustments become even less reliable (Bloj et al., 2016), and the problem is exacerbated. When CCI is used as the metric for color constancy, all such noise gets attributed to an inherent lack of constancy.

**Figure 16.**
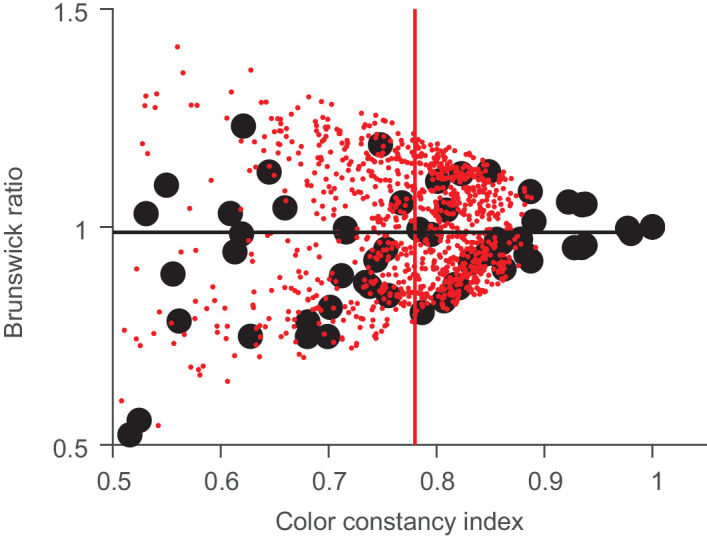
Relationship between BR and CCI. The black points are our data, and the small red points are simulations based on the color shifts from our study (23.84 ± 7.6 ∆*E*) and a noise error of 5.13 ∆*E* units. Note that the black circle at (1, 1) represents five data points that are completely overlapping. The black line represents the average BR, and the red line represents the average CCI for the simulated data.

The BR we used here first projects all measurements onto the axis of the colorimetric color change. This leads to an estimate that is unbiased in the face of measurement noise. Figure 16 illustrates this for our measurements. We plot both estimates, BR and CCI, against each other. The filled black symbols represent our data, and the small red dots represent simulated data of a perfectly color constant observer for the illumination shifts we used and a memory noise uniformly distributed within 5.13 ∆*E* units (the average ∆*E* in our study). Each red dot shows one color choice made by a hypothetical observer, who makes a perfect match in CIELAB color space. To that perfect match, a three-dimensional memory noise vector was added. The data and simulation show quite nicely that perfect color constancy in the presence of color matching noise leads to an unbiased BR estimate of close to 100%, (horizontal black line) whereas the CCI falls short of that, with an average that is close to 80% (vertical red line), a value that has been observed as the maximum in some previous studies. Note that the different indices cannot fully explain our results, because several data points have CCIs larger than 0.9. This basically never occurs in the simulated data, because it is exceedingly unlikely that the random noise vector is close to zero in all three dimensions.

Of course, any approach trying to condense a complex perceptual phenomenon into a single number has its flaws. The CCI is not unbiased in the face of sensory and memory noise. The BR can lead to high estimates even when the matches are far off, due to the projection. Its main advantage might lie in situations when constancy is very good and all of the selections or matches are close to the perfect match. This seems to have been the case in our study, where visual inspection of the data shows that there were no spuriously high values of the degree of constancy (Figure 12).

### Mechanisms

There are three good reasons why our results should not come as a surprise. First, they do agree with our experience of color in the natural world and with the use of color names to label objects. Second, even relatively simple computational models can achieve high levels of color constancy. This is evidenced by the fact that photographs taken under quite varying illumination conditions typically look correct when viewed in a different illumination context. The white balance algorithms built into most cameras achieve this feat. The magnitude of the correction easily becomes visible by turning off the automatic correction (if possible), as illustrated in Figure 1. Third, the neural mechanisms underlying color constancy are well known. There are relatively local adaptation mechanisms throughout the visual system, starting in the retina. There are global adaptation mechanisms emerging in higher cortical areas (Albers, Baumgartner, & Gegenfurtner, 2022; Bannert & Bartels, 2017). And, there are mechanisms for computing color contrast across edges as early as in primary visual cortex (Conway, 2001; Johnson, Hawken, & Shapley, 2001; Shapley & Hawken, 2011).

The importance of these local contrast edges has been emphasized numerous times. The very first computational model of constancy, the retinex algorithm by Edmund Land and John McCann (Land, 1964; Land & McCann, 1971), is based on such spatial comparisons. The influential work by David Foster and Sergio Nascimento (Foster & Nascimento, 1994; Nascimento, Ferreira, & Foster, 2002; Nascimento & Foster, 1997; Zaidi, Spehar, & DeBonet, 1997) pointed out very clearly that local cone excitation ratios could achieve an illumination-independent representation of the visual scene that preserves relations between different colors. There are numerous other potential mechanisms for achieving color constancy that have been summarized in review articles (Foster, 2011; Hurlbert, 2007; Smithson, 2005). Also, there is an abundance of computational models achieving color constancy at very high levels, ranging from geometric constraints (Uchikawa, Fukuda, Kitazawa, & MacLeod, 2012; Morimoto, Kusuyama, Fukuda, & Uchikawa, 2021) to deep neural networks (Flachot et al., 2019; Heidari-Gorji & Gegenfurtner, 2023). In light of all these different ways to achieve good color constancy, it should not come as a surprise that human observers do indeed exhibit good color constancy. Counter-examples can always be constructed, but how often we encounter such constructs in our natural environment can be debated.

## Conclusions

Our study resolves a riddle that has long puzzled vision scientists. In everyday life, we take color constancy for granted, whereas in the lab constancy turns out to be between mediocre and incomplete. We have shown that, in a natural environment using a natural task where the visual system has all possible cues available, we can indeed achieve near-perfection, as was shown decades ago for most other perceptual constancies, such as size constancy (Holway & Boring, 1941). This is reassuring in the light of a recent widely publicized example (#TheDress) showing a lack of constancy and large individual variation when insufficient cues about the illuminant and scene are available. Of course, our real-world stimuli are not very prone to experimental manipulation, as was the case in the earlier study by Kraft and Brainard (1999). Here, virtual reality offers an attractive option combining the best of natural environments and experimental possibilities. We have recently shown that the latest virtual reality systems can both produce photorealistic output while allowing accurate color calibration (Gil Rodríguez et al., 2022).

## Appendix

**Table A1.** Emission spectra of the five different illuminations in 1000*W/sr/m^2^.

Wavelength	Neutral	Reddish	Greenish	Bluish	Yellowish
380	1.7212	0.5497	0.8647	1.3072	0.5787
390	1.8831	0.6527	0.9191	1.4558	0.5648
400	1.9611	0.8966	0.9785	1.5354	0.5723
410	2.0650	1.1377	1.0424	1.6314	0.6069

**Table A1.** Continued.

Wavelength	Neutral	Reddish	Greenish	Bluish	Yellowish
420	2.2379	1.1894	1.1142	1.7776	0.6676
430	2.4882	1.2121	1.1900	1.9807	0.7923
440	2.6585	1.2991	1.2792	2.1069	1.0002
450	2.7547	1.4248	1.4283	2.1518	1.2247
460	2.8075	1.4700	1.5505	2.1392	1.4616
470	2.8805	1.4507	1.6345	2.1216	1.7029
480	2.9112	1.3892	1.6985	2.0468	1.9354
490	2.8833	1.3324	1.7394	1.9091	2.1523
500	2.8515	1.2582	1.7488	1.7559	2.3327
510	2.8173	1.1677	1.7246	1.5987	2.4663
520	2.7729	1.1182	1.6813	1.4314	2.5145
530	2.7356	1.1006	1.6231	1.2748	2.4558
540	2.6779	1.0745	1.5332	1.1279	2.3476
550	2.6243	1.0020	1.4158	1.0230	2.2108
560	2.5630	0.9606	1.2725	0.9717	2.0584
570	2.4962	1.0257	1.1252	0.9579	1.9068
580	2.4290	1.1876	0.9905	0.9625	1.7462
590	2.3553	1.3256	0.8642	0.9778	1.5872
600	2.2761	1.3814	0.7453	1.0140	1.4441
610	2.2206	1.4059	0.6454	1.0926	1.3190
620	2.1519	1.3704	0.5711	1.1852	1.2083
630	2.1030	1.3506	0.5198	1.2868	1.1007
640	2.0598	1.3303	0.4926	1.3860	1.0187
650	2.0043	1.3085	0.4884	1.4811	0.9569
660	1.9149	1.2960	0.5060	1.5504	0.9375
670	1.8601	1.3085	0.5348	1.6498	0.9430
680	1.8095	1.2210	0.5883	1.7299	0.9367
690	1.7167	1.1430	0.6575	1.7241	0.9316
700	1.6428	1.1225	0.7192	1.7023	0.9571
710	1.6018	1.0917	0.7364	1.6887	1.0001
720	1.5760	0.9821	0.8048	1.6739	0.9988
730	1.4970	0.9509	0.8526	1.5926	1.0178
740	1.4689	1.0492	0.9076	1.5569	1.0496
750	1.4393	1.0099	0.9683	1.5212	1.0493
760	1.4515	0.8417	1.0151	1.5244	1.0315
770	1.4795	0.7977	1.0399	1.5408	1.0446
780	1.4374	0.9760	1.0628	1.4929	1.0456

**Table A2.** All selections in Munsell notation. There were two repeats for each of the five illumination conditions.

Object	Neutral		Reddish		Greenish		Bluish		Yellowish	
Red book	7.5R4/16	7.5R4/14	7.5R4/16	7.5R4/14	7.5R4/14	7.5R4/14	7.5R4/16	7.5R4/14	7.5R4/14	7.5R4/14
Light pink scarf	2.5R7/6 2.5R7/8	10RP7/6	7.5RP7/6	7.5RP7/6	2.5R7/8	10RP7/8	2.5R8/4 2.5R7/6	10RP7/6 10RP7/4	7.5RP7/8	7.5RP7/8
Turqouise dummy	2.5BG6/10 2.5BG5/10	10G7/8 10G6/10	7.5G6/10	5G7/10	2.5BG6/10 10G6/10	2.5BG6/10	10G6/10	10G6/10 10G5/10	2.5BG5/10	10G5/10
Blue spike massage ball	5PB2/8	5PB2/8					5PB2/8	5PB3/10	5PB3/10	5PB3/10
Green t-shirt	10GY6/12 10GY7/10 10GY6/10	10GY6/10 10GY6/12 10GY5/10	10GY6/12	10GY6/10	10GY6/10	10GY6/10	10GY6/10 10GY6/12	10GY6/10 10GY6/12	2.5G5/10 2.5G6/10	2.5G5/10 2.5G6/10
Turqouise sweater	5BG7/8	2.5BG8/6	2.5BG8/6	2.5BG8/6	5BG7/6	2.5BG7/8	2.5BG7/8	2.5BG8/6	2.5BG8/6	7.5BG8/4
Green pick	10GY5/12	10GY5/10	10GY6/12	10GY6/10	7.5GY5/10	10GY5/12	10GY6/12	10GY6/12	10GY5/12	10GY5/12
Light yellow part of kitchen	5Y9/2	5Y9/2	7.5Y9/2	5Y9/2	7.5Y9/2	2.5Y9/2	7.5Y9/4	10Y9/2	5Y9/2	5Y9/2
Wooden chess piece	10YR6/10 10YR6/12	10YR7/14 7.5YR7/14	10YR7/12	10YR7/12 2.5Y8/16	10YR8/14	10YR7/14	10YR7/14 2.5Y8/14	10YR7/12 2.5Y8/16	10YR7/14 10YR8/14	10YR7/12 7.5YR7/6
Orange sweater	2.5YR6/16	2.5YR5/14	2.5YR6/14	2.5YR6/12	2.5YR6/16	2.5YR6/14	5YR6/14	2.5YR6/14	2.5YR6/14	2.5YR6/14
Brown sweater	10YR6/4 10YR5/4	7.5YR6/2	10YR6/2	10YR6/2	7.5YR6/2	10YR6/4	10YR6/4	10YR6/4	10YR6/2	10YR7/2
Green scarf	5GY5/4	5GY5/4	5GY6/6	2.5GY5/4	5GY5/6	5GY5/4	5GY5/4	5GY5/4	5GY5/4	5GY5/4
Red candle	5R3/10	5R3/10	7.5R4/16	7.5R4/16	7.5R4/16	7.5R4/16	7.5R3/12 5R3/10	7.5R4/14 5R4/14	7.5R3/12 5R3/10 7.5R3/10	10R3/10 7.5R3/12 7.5R3/10
Pink hippo	5RP7/10 5RP7/6	7.5RP6/12	5RP6/12	2.5RP7/10	5RP6/12	5RP7/10	5RP6/12	5RP7/10	5RP6/10	5RP6/10
Red metallic bike tool	7.5R4/16 5R4/14	2.5R4/14 5R4/12	2.5R4/14 5R4/14	10RP4/14 2.5R4/14	7.5R5/16	7.5R5/16 7.5R5/14	2.5R4/14	2.5R4/14	5R4/14 2.5R4/14	5R4/14 2.5R4/14
Blue elephant	7.5PB2/10	7.5PB2/10 5PB2/8	7.5PB2/10	7.5PB2/10	7.5PB2/10	5PB2/8	7.5PB2/10	7.5PB2/10	7.5PB2/10	5PB2/8
Turquoise potato	7.5BG5/10	7.5BG6/8	7.5BG7/8	10BG7/6	10BG6/8	10BG5/8	10BG6/8	10BG7/8	10BG6/8	10BG6/8
